# Clinical evaluation of a substitute of HLA-B*58:01 in different Chinese ethnic groups

**DOI:** 10.1590/1678-4685-GMB-2017-0258

**Published:** 2018-08-02

**Authors:** Xinju Zhang, Lei Jin, Zhiyuan Wu, Weizhe Ma, Yuming Chen, Gang Chen, Lixin Wang, Ming Guan

**Affiliations:** 1 Department of Central Laboratory, Huashan Hospital, Fudan University, Shanghai, China; 2 College of Allied Health Professions, Shanghai University of Medicine and Health Sciences, Shanghai, China; 3 Department of Laboratory Medicine, North Huashan Hospital, Fudan University, Shanghai, China; 4 Department of Laboratory Medicine, Huashan Hospital, Fudan University, Shanghai, China; 5 Department of Geriatrics, Huashan Hospital, Fudan University, Shanghai, China; 6 The Medical Laboratory Center of General Hospital of Ningxia Medical University, Yinchuan, China; 7 The Medical Laboratory of Cardio-Cerebral Vascular Disease Hospital of General Hospital of Ningxia Medical University, Yinchuan, China; 8 Ningxia Key Laboratory of Clinical Pathogens, Yinchuan, China

**Keywords:** rs9263726, HLA-B*58:01, allopurinol hypersensitivity reaction, tag SNP

## Abstract

The goal of this research was to investigate the linkage disequilibrium between rs9263726 and HLA-B*58:01 in different Chinese ethnic groups (Han, Tibet, and Hui) and to study the feasibility of rs9263726 replacing HLA-B*58:01 as an efficient indicator of potential allopurinol hypersensitivity syndrome. In this study, rs9263726 and HLA-B*58:01 were detected in all samples. For samples of individuals whose rs9263726 genotypes were not consistent with HLA-B*58:01, we did high-resolution typing of HLA-B gene to further confirm the correlation of rs9263726 genotype and special HLA-B alleles. We confirmed that the linkage disequilibrium between rs9263726 and HLA-B*58:01 was more significant in the Han ethnic group (r^2^=0.886, D’=1.0) than in the Tibet and Hui ethnic groups (for Tibetan, r^2^=0.606, D’=0.866; for Hui, r^2^=0.622, D’=0.924). For Han Chinese, samples with the GG genotype of rs9263726 did not carry HLA-B*58:01, while AA genotype samples were homozygous carriers of HLA-B*58:01. However, GA genotype samples of rs9263726 required a more sophisticated HLA-B genotyping assay before it was possible to identify whether they were HLA-B*58:01 carriers or not. For Tibetan and Hui, the linkage disequilibrium between rs9263726 and HLA-B*58:01 was not significant. Therefore, rs9263726 cannot replace HLA-B*58:01 in these two groups.

## Introduction

Allopurinol is recommended as the first-line pharmacologic urate-lowering therapy in gout. However, severe hypersensitivity of some patients to allopurinol limits its use in clinical medicine. HLA-B*58:01 has been recommended as marker of the severe allopurinol hypersensitivity syndrome in some subpopulations with high prevalence of the HLA-B*58:01 allele, including Chinese, European, Italian, Korean, and Thai (Pavlos *et al.*, 2012). However, because of the complexity of the HLA-B polymorphic loci, the current detection methods, such as PCR-SSP and PCR-SSO, are too complex to operate, and several interference factors make the detection of HLA-B*58:01 allele unfeasible for wide application in the clinic.

Tohkin *et al.* (2013) reported that rs9263726 was in absolute linkage disequilibrium (LD) with HLA-B*58:01 in a Japanese population and could be used as a surrogate marker of HLA-B*58:01 to predict patients with high-risk of severe allopurinol hypersensitivity syndrome. However, unlike the results for this Japanese population, a recent study showed a lack of LD between the rs9263726 A allele and HLA-B*58:01 in Australians (Vidal *et al.*, 2016). This observation suggests that rs9263726 frequencies may differ across different ethnic groups.

In this study, we investigated the linkage relationship between rs9263726 and HLA-B*58:01 in three Chinese populations. In light of the vast territory, large number of ethnic minorities, and genetic differences in China, this study analyzed the feasibility of substituting HLA-B*58:01 for rs9263726 in Tibetan, Han, and Hui Chinese people based on the morbidity of hyperuricemia. Our data demonstrate that rs9263726 can substitute for HLA-B*58:01 as marker of the severe allopurinol hypersensitivity syndrome in Han Chinese, but not in Tibetan and Hui people.

## Subjects and Methods

### Subjects

For this study, samples were randomly collected from three groups of participants from different regions of China. The first group consisted of 454 healthy South Han Chinese who were volunteers for clinical physical examination and 205 gout Han patients seen in Huashan Hospital between January 2013 and December 2015. The second group consisted of 500 healthy Tibetans residing in Xining Region of Qinghai Province, recruited from the Tibetan Medical Hospital of Qinghai Province. The third group was composed of 200 healthy Hui people residing in Ningxia Hui Autonomous Region, who were recruited from the General Hospital of Ningxia Medical University. Qinghai Province and the Ningxia Hui Autonomous Region have retained comparatively intact genetically isolated populations. Due to differences in diet structure, climate, and genetic variation, the incidence of hyperuricemia in Han people is higher than that in Hui people (Cong *et al.*, 2009), but lower than in Tibetans (Cong *et al.*, 2009). Therefore, we chose these three ethnicities for the gene analysis. The healthy individuals did not have a personal or familial history of hyperuricemia or gout. This study was approved by the Ethics Committee of Huashan Hospital and conducted in accordance with the Declaration of Helsinki guidelines for ethics in research. Written informed consent was obtained from each patient prior to collection of the specimens.

### Sample preparation

During transportation, whole blood samples were stored in dry ice to prevent DNA degradation. Genomic DNA was extracted from EDTA-anticoagulated whole-blood samples using the QIAamp DNA Blood Mini Kit (Qiagen, Hilden, Germany) and kept at -20 °C.

### rs9263726 genotyping by HRM with an unlabeled probe method

We detected rs9263726(G > A) alleles in all samples by high-resolution melting (HRM) analysis with an unlabeled probe method using the positive and negative control samples identified by Sanger sequencing. The forward and reverse primers were 5’-ACCCCAGCTTTACA AGGACCC-3’ and 5’-GCTCCATGTGGCAAAGTCGG TCA-3’, respectively, and the unlabeled probe was 5’-CTCCGAGGAAACTCATCCCCCC-PHO-3’. The reagent mix was made up as follows: 10 μL Premix *Taq* HS (Takara, Japan), 0.5 μL of forward primer (2 μM), 0.5 μL of reverse primer (10 μM), 0.5 μL of unlabeled probe (10 μM) and 25 ng DNA that was added to the solution before completing with water to 20 μL. The PCR conditions were: initial denaturation at 95 °C for 2 min, 50 cycles at 95 °C for 30 s, 55 °C for 30 s, and 72 °C for 30 s. After amplification, 0.6 μL of 1 × SYTO 9 dye (Invitrogen, Carlsbad, California) was added. The reaction tubes were then transferred to a Rotor Gene Q system (Qiagen) and processed for HRM analysis. The PCR products were heated to 95 °C for 1 min followed by rapid cooling to 40 °C for 1 min to facilitate heteroduplex formation. Melting curve analysis was performed by raising the temperature from 55 °C to 89 °C at 0.5 °C /s. Genotypes were identified by the melting temperatures indicated by peaks on the derivate plots. To confirm the results, all samples with the AA genotype, and 40 randomly selected ones with the GA and 30 with the GG genotype were examined by Sanger sequencing.

To raise the accuracy of the developed method and to avoid interference by other SNPs, we screened the interference sites surrounding rs9263726. Fifty samples from healthy Han people’s DNA were randomly selected for sequencing from 200 bp upstream to 200 bp downstream of the rs9263726 allele and construction of a database of distribution of polymorphic loci and common mutations in the area surrounding the rs9263726 allele. The sequencing service was provided by Shanghai Jie Li Biological Technology Co., Ltd (Shanghai, China).

### HLA-B*58:01 typing by TaqMan assay

HLA-B*58:01 typing of all samples was done by a method previously developed in our laboratory (Zhang *et al.*, 2015). We compared the HLA-B*58:01 with the rs9263726 genotype to analyze the correlation of HLA-B*58:01 carrier status and rs9263726 genotypes (GG, GA, and AA).

### High-resolution genotyping of the HLA-B allele

To further confirm the correlation between the rs9263726 genotype and special HLA-B alleles, we performed high-resolution typing of the HLA-B gene by using the LABType® HD B Locus Typing Test kit (One Lambda, USA) in samples of individuals whose rs9263726 genotypes were not consistent with HLA-B*58:01. Following the instructions of the LABType® kit, we detected the binding to magnetic beads and amplicons on a Luminex 200 machine, and then imported the data to HLA Fusion software 3.0.0 to analyze the HLA-B high-resolution gene typing.

### Statistical analysis

Statistical analysis was done using STATA 10.0 (StataCorp LLC, USA). Chi-squared tests were used to detect frequency differences of HLA-B*58:01 in Han, Tibetan, and Hui people. The distribution of the rs9263726 genotype in these three ethnicities was analyzed using SHEsis online software (http://analysis.bio-x.cn/SHEsisMain.htm). D’ and r^2^ > 0.8 were considered as indicating the existence of a strong LD. A *p-*value < 0.05 was considered as statistically significant.

## Results

### rs9263726 genotyping with the unlabeled probe HRM method

**Figure 1:**
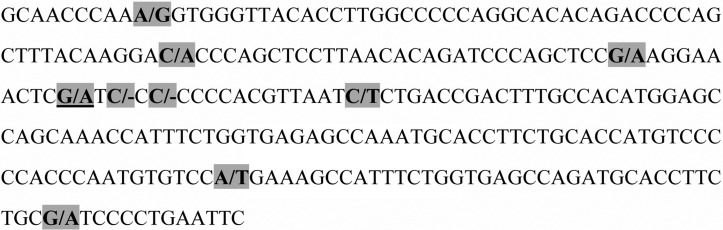
Distribution of common SNPs and indels within the range from 99 bp upstream to 156 bp downstream of rs9263726. The underlined bases are the position of rs9263726 (G > A). Bases with gray shading represent the indel or SNP and its allele change.

The sequencing results indicated that a large number of SNPs and deletions were dispersed around the rs9263726 allele (Figure 1). The closest SNP was just 2 bases from rs9263726, after which there were 7 continuous cytosine bases from the second base.

Because of the interference from these SNPs and deletions, the results of the common Taqman probe method were disturbed by misidentification and/or a nonspecific interference signal while genotyping rs9263726. Therefore, in this study, we decided to detect rs9263726 with an unlabeled probe-HRM method, which could identify the GG, GA, and AA genotypes in a single amplification reaction.

**Figure 2:**
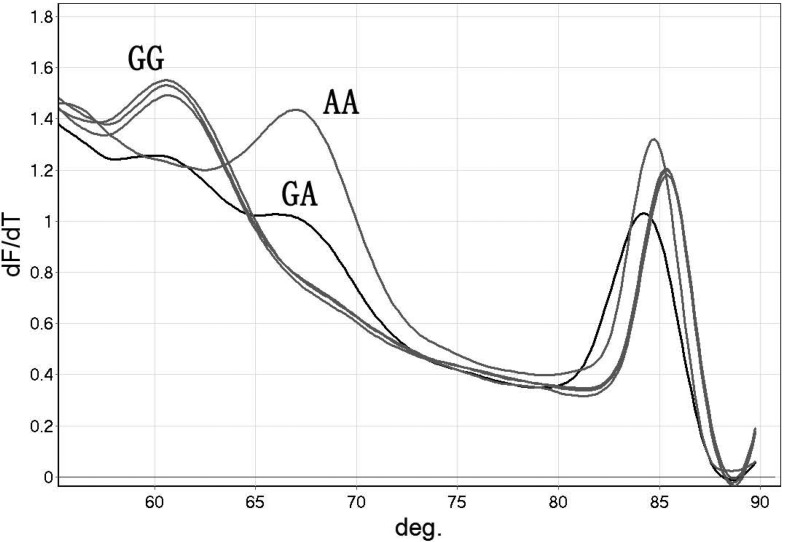
Detection of rs9263726 using an unlabeled probe-HRM. Melting peaks lower than 72 °C represent a probe/product duplex. Other melting peaks higher than 80 °C represent a product/product duplex.

By this method, the rs9263726 genotypes were confirmed according to the melting temperature of the amplicons. Each sample had two melting peaks. Melting peaks lower than 72 °C were used to examine base changes in the area covered by the probe; melting peaks higher than 80 °C were used to examine base changes in the whole amplification area. For probe/product duplexes, the melting peaks on the left with a lower melting temperature (about 60.7 °C) represent the GG genotype and melting peaks on the right with a higher melting temperature (about 67 °C) represent the AA genotype; both melting peaks appeared in the GA genotype (Figure 2).

In this study that included 454 Han, 500 Tibetan, and 200 Hui people, the distribution of rs9263726 genotypes showed significant differences among the three Chinese populations (*p*=0.0004). Genotype and allele frequences are shown in Table 1. The SNP tested was at Hardy-Weinberg equilibrium in all three groups.

**Table 1:** Allele and genotype frequencies of rs9263726 in Han, Tibetan, and Hui people.

Population	rs9263726			*p-*value	Allele		*p-*value
	GG	GA	AA		G	A	
Tibetan people (n=500)	463(0.926)	36(0.072)	1(0.002)	0.0004	962(0.96)	38(0.04)	< 0.0001
Hui people (n=200)	162(0.810)	36(0.180)	2(0.010)		360(0.90)	40(0.10)	
Han people (n=454)	399(0.879)	51(0.112)	4(0.009)		849(0.93)	59(0.07)	
Han patients with gout (n=205)	180(0.878)	25(0.122)	0(0.000)	0.3831^#^	385(0.94)	25(0.06)	0.7830^#^

# Compared with Han people.

### Frequency of HLA-B*58:01 allele carriers in Han, Tibetan, and Hui people

We also investigated the frequency of HLA-B*58:01 allele carriers in the Han, Tibetan, and Hui groups by the method we had previously developed (Zhang *et al.*, 2015). We found that the positive rate of HLA-B*58:01 in Han, Tibetan, and Hui people was 10.6%, 6.0%, and 15.0%, respectively (Table 2). The frequency of HLA-B*58:01 allele carriers was significantly different for the three ethnic groups (Tibetan vs Hui, *p*=0.0001;Tibetan vs Han, *p*=0.0100;Han vs Hui, *p*=0.1075).

**Table 2:** Performance of rs9263726 to tag HLA-B*58:01.

Group	HLA-B*58:01	rs9263726		Sensitivity	Specificity	Positive predictive value	Negative predictive value	r^2^	D’	HLA-B*58:01	
		AA+GA	GG							Positive rate	*p-*value
Tibetan	-	10	460	90.0%	97.9%	73.0%	99.4%	0.606	0.866	6.0%	^#^
	+	27	3								
Hui	-	10	160	93.3%	94.1%	73.7%	98.8%	0.622	0.924	15.0%	0.0001^#^
	+	28	2								
Han	-	7	399	100%	98.3%	87.3%	100%	0.886	1.000	10.6%	0.0100^#^
	+	48	0								
Han patients with gout	-	4	180	100%	97.8%	84.0%	100%	0.831	1.000	10.2%	0.8985^*^
	+	21	0								

# Compared with the positive rate of HLA-B*58:01 in Tibetan People.

* Compared with the positive rate of HLA-B*58:01 in Han People.

### Performance of rs9263726 to tag HLA-B*58:01

Performance of rs9263726 to tag HLA-B*58:01 was assessed by its positive predictive value (PPV), negative predictive value (NPV), sensitivity, and specificity. PPV is given as true positive/(true positive + false positive), NPV is true negative/(true negative+false negative), sensitivity is true positive/(true positive+false negative), and specificity is true negative/(true negative+false positive). We hypothesized that the GG genotype of rs9263726 represents a non-HLA-B*58:01 carrier, and that the GA or AA genotypes both represent an HLA-B*58:01 carrier. The NPV of rs9263726 in Han people was 100% because all samples with the GG genotype of rs9263726 were non-HLA-B*58:01 carriers. Sensitivity was also 100% because all HLA-B*58:01 carriers were GA or AA genotypes of rs9263726. However, PPV was only 87.3% because there were 7 out of 55 samples with the GA or AA genotypes of rs9263726 that were non-HLA-B*58:01 carriers. Specificity was 98.3% because 7 out of 406 were non-HLA-B*58:01 carriers with GA or AA genotype of rs9263726. The NPV and PPV in Tibetan people were 99.4% and 73.0%, respectively; in Hui people, they were 98.8% and 73.7%, respectively. The performance of rs9263726 to tag HLA-B*58:01 is shown in Table 2.

### Validation of the performance of rs9263726 to tag HLA-B*58:01 in Han patients with gout

The genotypes of rs9263726 and HLA-B*58:01 in 205 Han patients with gout were investigated to detect their relationship in gout patients. The distribution of rs9263726 and the correlation with HLA-B*58:01 in gout patients were similar to that seen in the healthy Han population (Tables 1 and 2).

### Genotyping of HLA-B and rs9263726 in samples with inconsistent results

For samples whose rs9263726 genotypes were not linked with the HLA-B*58:01 carrying status, we used the LABType® HD B Locus Typing Test for high-resolution typing of the HLA-B allele and to further analyze the linkage relationship between HLA-B*58:01 and rs9263726 in the three Chinese populations. In our study, HLA-B*58:01 carriers with the rs9263726 GG genotype did not have exactly the same HLA-B alleles in Tibetan and Hui people. In the 500 Tibetan subjects there were three HLA-B*58:01 carriers with the rs9263726 GG genotype: B*51:01/B*58:01, B*51:01/B*58:01, and B*44:03/B*58:01. In Hui samples, two of the 200 subjects were HLA-B*58:01 carriers with the GG genotype of rs9263726: B*51:01/B*58:01 and B*40:01/B*58:01 (Table 3). No HLA-B*58:01 carriers with the GG genotype of rs9263726 were found in the Han population in this study, and all HLA-B*51:01/B*58:01 and HLA-B*40:01/B*58:01 carriers were of the GA genotype.

**Table 3:** HLA-B genotyping for HLA-B*58:01 carriers with the GG genotype of rs9263726.

Population	Sample No.	HLA-B genotyping	rs9263726
Tibetan	ZM56	51:01/58:01	G G
	ZM170	51:01/58:01	G G
	ZM267	44:03/58:01	G G
Hui	HM103	51:01/58:01	G G
	HM196	40:01/58:01	G G

### Correlation analysis between the AA genotype and HLA-B*58:01 homozygotes

The frequency of AA homozygotes for rs9263726 in the Tibetan, Han, and Hui populations was 0.2%, 0.9% and 1.0%, respectively (Table 1). The samples for AA homozygote were analyzed using high-resolution typing of HLA-B by the LABType® HD B Locus Typing Test Kit. All of the four AA genotype samples were HLA-B*58:01/HLA-B*58:01 in the Han population; the two AA genotype samples in the Hui population were B*44:03/B*58:01; and the only AA homozygote in the Tibetan population was 58:01/58:01 (Table 4). There was a significant difference in the linkage between the AA genotype and HLA-B*58:01 homozygotes in these three ethnic groups.

**Table 4:** HLA-B genotyping of AA homozygote samples in the three populations.

Population	Sample No.	HLA-B genotyping	rs9263726
Tibetan	ZM480	58:01/58:01	A A
Han	HS64	58:01/58:01	A A
	HS66	58:01/58:01	A A
	HS180	58:01/58:01	A A
	HS188	58:01/58:01	A A
Hui	HM49	44:03/58:01	A A
	HM127	44:03/58:01	A A

## Discussion

The American College of Rheumatology (ACR) recommends that HLA-B*58:01 screening should be considered a risk management component in Chinese patients before taking allopurinol (Khanna *et al.*, 2012; Cheng *et al.*, 2015; Ko *et al.*, 2015). However, because of the high polymorphism and complexity of HLA-B alleles, there are numerous technical bottlenecks to overcome when extending HLA-B*58:01 detection from scientific research to clinical applications (Guo *et al.*, 1999). Even though Sanger sequencing could identify the base sequence in most cases, it still leads to ambiguous results in HLA-B genotyping. Therefore, there is a need to find a tag SNP in HLA-B*58:01 for speeding up its application in the clinic. Compared with the HLA typing method, single SNP detection is more convenient and cost-effective.

As some alleles within nearby genes may be in LD with each other, a tag SNP could be found through this way. The PSORS1C1 (Psoriasis susceptibility 1 candidate 1) gene is only 215 kb away from the HLA-B gene, and rs9263726 (110G > A, Arg37His), which is located in the PSORS1C1 gene, was found in complete LD with HLA-B*58:01 (r^2^=1, D’=1) in a Japanese population (Maekawa *et al.*, 2012; Tohkin *et al.*, 2013). However, the LD might differ significantly between different populations. In fact, a lack of LD between rs9263726 and HLA-B*58:01 was found in an Australian population (Vidal *et al.*, 2016).

In this study, we investigated the LD between rs9263726 and HLA-B*58:01 in Han, Tibet, and Hui populations in China. The degree of LD in the Han population (r^2^=0.886, D’=1.0) was higher than in the Tibetan (r^2^=0.606, D’=0.866) and Hui populations (r^2^=0.622, D’=0.924), which means that rs9263726 could be used as a tag SNP of HLA-B*58:01 in Han Chinese but not in the other two populations. Also, in Han patients with gout, the LD between rs9263726 and HLA-B*58:01 was similar to that seen in the healthy Han population. Moreover, all Han subjects with the rs9263726 GG genotype were non-HLA-B*58:01 carriers, which means that the NPV was 100%. The clinical significance of HLA-B*58:01 is that non-HLA-B*58:01 carriers will not suffer from allopurinol-induced severe cutaneous adverse drug reactions. Therefore, rs9263726, which has an NPV of 100%, can be used as a surrogate marker of HLA-B*58:01 in clinical diagnosis for allopurinol hypersensitivity syndrome.

In contrast, four of the 454 samples of healthy Han (0.9%) were AA genotype of rs9263726, and all were homozygous carriers of HLA-B*58:01. Although it is still unclear whether there is any difference in the severity and rapidity of cutaneous adverse drug reactions between HLA-B*58:01 homozygous and heterozygous carriers after they take allopurinol, there are three hypotheses about how T-cell receptors and HLA interact with drugs and peptides (Hershfield *et al.*, 2013; Yun *et al.*, 2014), one suggesting that an increase of peptide-binding clefts may accelerate or intensify the hypersensitivity. However, this hypothesis requires further investigation. In comparison, such linkage between the AA genotype of rs9263726 and homozygous carriers of HLA-B*58:01 was not found in the Hui population. Two out of the 200 Hui group samples (1.0%) were AA genotype of rs9263726, and both of them were from carriers of HLA-B*44:03/B*58:01. There was only one sample with AA genotype in the Tibetan population (0.2%). Although the sample was from a HLA-B*58:01/B*58:01 carrier, the result is not sufficient to provide support for such a relationship. Moreover, five samples from the Tibet and Hui populations that were categorized as “false negative” displayed different results in HLA-B typing (Table 3), also indicating that the degrees of LD are weak between rs9263726 and HLA-B*58:01 in both Hui and Tibet populations.

Liu *et al.* (2015) detected SNPs around the HLA-B gene in 880 Han Chinese by using Illumina OmniExpress BeadChip assays and discovered seven SNPs with a sensitivity of 100% and a specificity ≥ 95% that were able to tag HLA-B*58:01, but exclusive of rs9263726. This might be explained by sample differences. In their study, rs9263726 may be rejected due to lack of sufficient specificity. However, in the seven reported SNPs, the highest values of PPV, sensitivity, and specificity were 76.4%, 100%, and 97.41%, respectively, all of which are lower than or equal to 87.3%, 100%, and 98.3% for rs9263726 in this study. Therefore, rs9263726 may be a more suitable surrogate marker of HLA-B*58:01. Kang *et al.* (2016) also investigated the correlation of rs9263726 and HLA-B*58:01, but their distribution and frequency of rs9263726 in the Han population was different from ours. They did not find the GA genotype of rs9263726, for which the incidence was as high as 11.2% in our study, and the linkage between rs9263726 and HLA-B*58:01 was not as obvious as in our study. These differences might be explained by hereditary differences present in South and West China (Xu *et al.*, 2009).

Before establishing a method to genotype rs9263726, we determined the distribution of interference sites surrounding rs9263726 through Sanger sequencing in the range from 200 bp upstream to 200 bp downstream. We found many SNPs and/or mutations (variants) around the allele. There were only two bases from the rs9263726 allele to the closest mutation site, and from the second base after the allele there were seven continuous cytosine bases. With these interference sites, the common Taqman probe method would fail to genotype rs9263726 because of misidentification or a nonspecific interference signal. Therefore, we developed an unlabeled probe-HRM method to distinguish the GG, GA, and AA genotypes in a single amplification reaction. The accuracy of the developed method can be considered as high, even though there may be unknown new mutations in the detection area that could reflect in a change in the melting curve.

In conclusion, we report that rs9263726 could be used as a surrogate marker of HLA-B*58:01 to predict potential allopurinol hypersensitivity reaction prior to initiation of allopurinol in Han Chinese. Han people with the GG genotype of rs9263726 represent non-HLA-B*58:01 carriers and could take allopurinol without high risk of a hypersensitivity reaction. Individuals with the AA genotype represent homozygous HLA-B*58:01 carriers and an alternative to allopurinol should be prescribed because they are at high risk for severe allopurinol hypersensitivity reaction. However, individuals with the GA genotype require further complex detection in HLA-B genotyping to identify whether they are HLA-B*58:01 carriers. The disequilibrium in the linkage between rs9263726 and HLA-B*58:01 is too low to have statistical significance in Hui and Tibet populations, so rs9263726 cannot replace HLA-B*58:01 in these two populations.
